# 

**Published:** 1847-01-01

**Authors:** 


### MEDICO-CHIRURGICAL

### REVIEW,

AND

### JOURNAL OF PRACTICAL MEDICINE.

v. [46]

---

NEW SERIES.

VOL. V.

---

OCTOBER 1, 1846, TO MARCH 31, 1847.

Surgeon General's Office LIBRARY 57488 Washington, D.C.

LONDON:

SAMUEL HIGHLEY, 32, FLEET STREET,

1847.

WI
ME 75 T

PRINTED BY F. HAYDEN,
Little College Street, Westminster.

### BIBLIOGRAPHICAL RECORD.

1. Guy's Hospital Reports. Second Series. Edited by *George Hilaro Barlow*, M.D., *Edward Cock, Edmund L. Birkett*, M.B., and *Alfred Poland.* With Plates. Vol. IV. Octavo, pp. 502. London, 1846.

2. A Descriptive Catalogue of the Anatomical Museum of St. Bartholomew's Hospital. Published by Order of the Governors. Vol. I., containing the Descriptions of the Specimens illustrative of Pathological Anatomy. Royal 8vo, pp. 503. London, 1846.

*An admirable precedent to the Governors of other hospitals.*

3. Records of Harvey, in Extracts from the Journals of the Hospital of St. Bartholomew's. Published by permission of the President and Treasurer, with Notes by *James Paget.* 8vo, pp. 51. London, 1846.

*Will be useful to future biographers of this great man.*

4. Report of the Medical Officers of the Lunatic Asylum for the County of Lancaster, instituted 28th July, 1816. 8vo, pp. 16. Lancaster, 1846.

5. A Review of Homœopathy, Allopathy, and Young Physic. By *L. M. Lawson,* M.D. 8vo, pp. 34. Lexington, U.S. 1846.

6. Practical Observations and Suggestions in Medicine. Second Series. By *Marshall Hall,* M.D. 12mo, 372. London, 1846.

7. Urinary Deposits, their Diagnosis, Pathology, and Therapeutical Indications. By *Golding Bird,* M.D. Second Edition. 12mo, pp. 380. London, 1846.

8. The Moral Aspect of Medical Life, consisting of the Akesios of Professor *K. F. H. Marx.* Translated from the German, with Biographical Notices and illustrative Remarks. By *James Mackness,* M.D. 12mo, pp. 362. London, 1846.

9. A Guide for the proper Treatment of the Teeth, with a View to their Preservation; containing a popular Explanation of their Structure and Appendages, with Directions for the Management of them in Health, together with Observations on the best Means of replacing them when lost. By *W. Keneely Bridgman.* Foolscap 8vo, pp. 88. London, 1846.

*Sensibly written, but addressed too much to the general public.*

10. Chemistry and Physics, in relation to Physiology and Pathology. By Baron *Justus Liebig,* M.D. 8vo, pp. 116. London, 1846.

*A valuable Essay. It forms a portion of the author's Animal Chemistry, reviewed in our present number.*

11. Chemistry of the Four Seasons—Spring, Summer, Autumn, and Winter. An Essay principally concerning Natural Phenomena, admitting of Interpretation by Chemical Science, and illustrating Passages of Scripture. By *Thomas Griffiths.* Small 8vo, pp. 515. London, 1846.

12. On the Pathology and Treatment of Scrofula; being the Forthergillian Prize Essay for 1846. By *Robert Mortimer Glover,* M.D. 8vo, pp. 327, Plates. London.

13. Animal Chemistry, or Chemistry in its Application to Physiology and Pathology. By Baron *Liebig.* Edited by *William Gregory,* M.D. Third Edition, revised and greatly enlarged. Part I. The Chemical Process of Respiration and Nutrition—the Metamorphosis of Animal Tissue. § 1. Method to be pursued in the Investigation. 8vo, pp. 282. London.

14. Lectures and Observations on Clinical Surgery. By *Andrew Ellis.* 8vo, pp. 287. Dublin. *In our next.*

15. On Medical Education; being a Lecture delivered at King's College, London, at the Opening of the Medical Session, 1846-7; to which is added, a Lecture delivered on the same Occasion in the Year 1842. By *William Augustus Guy,* M.B. 8vo, pp. 64. London.

*Contains much judicious advice to medical students.*

16. The Microscopic Anatomy of the Human Body in Health and Disease. Illustrated with numerous Drawings in Colour. By *Arthur Hill Hassall.* Parts III., IV., and V. London.

17. The Pathological Anatomy of the Human Body. By *Julius Vogel,* M.D. Translated from the German, with additions, by *George E. Day,* M.A. and L.M. Illustrated with upwards of one hundred plain and coloured Engravings. 8vo, pp. 614. London.

18. Hand-Book of Human Anatomy, general, special, and topographical. Translated from the Original German of Dr. *Alfred Von Behr,* and adapted to the Use of the English Student. By *John Birkett,* Pp. 457.

19. Elements of Chemistry, including the Applications of the Science in the Arts. By *Thomas Graham,* F.R.S. Second Edition, entirely revised and greatly enlarged. With additional Wood Engravings. Part I. 8vo, pp. 160. London.

20. Seventh Annual Report of the Registrar-General of Births, Deaths, and Marriages in England—Abstracts of the Years 1843-4. 8vo, pp. 347. London.

21. A Manual of the Diseases of the Eye, or Treatise on Ophthalmology. By *S. Littel,* Jun. M.D. Second Edition, revised and enlarged. 8vo, pp. 384. Philadelphia.

22. Practical Remarks on Near-Sight, Aged Sight, and Impaired Vision; with Oservations on the Use of Glasses, and on Artificial Light. By *William White Cooper.* 8vo, 226. London.

23. A Reply to the Review of Dr. Drummond's First Steps to Anatomy, contained in the British and Foreign Medical Review for April, 1846. 8vo, pp. 31. Belfast.

24. Principles of Human Physiology, with their chief Applications to Pathology, Hygiene, and Forensic Medicine. By *William B. Carpenter*, M.D. Third Edition. 8vo, pp. 800. London.

25. A Manual of Materia Medica and Therapeutics, including the Preparations of the Pharmacopœias of London, Edinburgh, and Dublin, with many new Medicines. By *T. ForbesRoyle*, M.D. 8vo, pp. 729. London.

26. Observations on Hydropathy, with an Account of the Principal Cold Water Establishments of Germany. By *J. Stevenson Bushnan*, M.D. 12mo, pp. 182. London.

27. Liebig's Question to Mulder, tested by Morality and Science. By Dr. *G. T. Mulder*. Translated by Dr. *P. F. H. Fromberg*. 8vo, pp. 22. London.

28. Lectures on Subjects connected with Clinical Medicine, comprising Diseases of the Heart. By *P. M. Latham*, M.D. Vol. II. 8vo, pp. 428.

29. A System of Surgery. By *J. M. Chelius*. Translated from the German, and accompanied with additional Notes and Observations. By *John F. South*. Part 14. 8vo. London.

30. Synopsis of the Physical Signs of the Diseases of the Lungs. By *James Turnbull*, M.D.

31. Medico-Chirurgical Transactions Vol. XI. of the New Series.

32. The Potato-Plant; its Uses and Properties, together with the Cause of the Present Malady. The Extension of that Disease to other Plants, the Question of Famine arising therefrom, and the best Means of averting that Calamity. By *Alfred Smee*, F.R.S. Illustrated with Ten Lithographs. 8vo, pp. 168. Longman, 1846.

33. Life of George Cheyne, M.D., with Extracts from his Works and Correspondence. 18mo, pp. 141. London, 1846.

34. On Diseases of the Skin. By *Erasmus Wilson*, F.R.S. Second Edition. 8vo, pp. 526. Eight coloured Plates. London, 1847.

35. A Treatise on the Plague, more especially on the Police Management of that Disease. Illustrated by the Plan of Operations successfully carried into Effect in the late Plague of Corfu. With Hints on Quarantine. By *A. White*, M.D. 8vo, pp. 355. London, 1846.

*In our next.*

36. The Medical Examiner, and Record of Medical Science. Edited by *Rob. M. Huston*, M.D. For June, 1846. Philadelphia.

37. Revue Medicale, from May to August, 1846. *In exchange.*

38. Dublin Medical Review, No. 4. *In exchange.*

39. Edinburgh Medical and Surgical Journal, for October. *In exchange.*

40. British and Foreign Medical Review, for October. *In exchange.*

41. Medical Gazette, October to January. *In exchange.*

42. Gazette Medicale, October to January. *In exchange.*

43. The American Journal of the Medical Sciences. Edited by *Isaac Hays*, M.D. *In exchange.*

---

### NOTICE TO CORRESPONDENTS, &c.

The reference made in our last number, page 488, to a volume of Knight's Weekly Series, should have been to Dr. Lankester's Lectures.

The reader is requested to substitute the figures 20 for 30 at page 493, line 10 from bottom.

We have received a letter from Mr. Parkin, complaining of our notice of his pamphlet on Cholera. Any reader, who will take the trouble to refer to Dr. Johnson's review of the first edition of it in No. 50 of this Journal, may convince himself of the justice of our strictures.

The press of matter this quarter has unexpectedly prevented the insertion of a review of Vogel's Pathological Anatomy, which is in type. We are also compelled to defer notices of Humboldt's Kosmos, Henfrey's Structural and Physiological Botany, Lee's Observations on Mineral Waters, and Gui Patin's Letters, edited by M. Reveillé-Parise.

